# Observing Virtual Arms that You Imagine Are Yours Increases the Galvanic Skin Response to an Unexpected Threat

**DOI:** 10.1371/journal.pone.0003082

**Published:** 2008-08-28

**Authors:** Karin Hägni, Kynan Eng, Marie-Claude Hepp-Reymond, Lisa Holper, Birgit Keisker, Ewa Siekierka, Daniel C. Kiper

**Affiliations:** 1 Institute of Human Movement Sciences and Sport, ETH Zurich, Zurich, Switzerland; 2 Institute of Neuroinformatics, University of Zurich and ETH Zurich, Zurich, Switzerland; 3 Institute of Neuroradiology, University Hospital Zurich, Zurich, Switzerland; 4 Department of Neurology, University Hospital Zurich, Zurich, Switzerland; Harvard Medical School, United States of America

## Abstract

Multi-modal visuo-tactile stimulation of the type performed in the rubber hand illusion can induce the brain to temporarily incorporate external objects into the body image. In this study we show that audio-visual stimulation combined with mental imagery more rapidly elicits an elevated physiological response (skin conductance) after an unexpected threat to a virtual limb, compared to audio-visual stimulation alone. Two groups of subjects seated in front of a monitor watched a first-person perspective view of slow movements of two virtual arms intercepting virtual balls rolling towards the viewer. One group was instructed to simply observe the movements of the two virtual arms, while the other group was instructed to observe the virtual arms and imagine that the arms were their own. After 84 seconds the right virtual arm was unexpectedly “stabbed” by a knife and began “bleeding”. This aversive stimulus caused both groups to show a significant increase in skin conductance. In addition, the observation-with-imagery group showed a significantly higher skin conductance (p<0.05) than the observation-only group over a 2-second period shortly after the aversive stimulus onset. No corresponding change was found in subjects' heart rates. Our results suggest that simple visual input combined with mental imagery may induce the brain to measurably temporarily incorporate external objects into its body image.

## Introduction

In the rubber hand illusion (RHI) [1]–[3], subjects watching a rubber arm being stroked with a brush, while being simultaneously stimulated in the same way, quickly incorporate the rubber arm into their body image. This illusion is strong enough to elicit a cortical anxiety response when the rubber arm is threatened, which is measurable using functional magnetic resonance imaging (fMRI) [4]. The traditional RHI protocol involves stimulating a real rubber arm to create the feeling of ownership. Is such a rather cumbersome stimulation protocol always necessary? Could simple movement observation coupled with motor imagery also induce measurable ownership of an external limb?

The process of movement observation and imagery plays a key role in the mirror neuron system. Mirror neurons, first discovered in monkeys [5]–[7] and later postulated to exist in the human brain [8], are active under three conditions: i) when observing a movement performed by a conspecific [9], ii) when executing a similar movement [10], or iii) when imagining the movement [11]. A functional magnetic resonance imaging (fMRI) study employing the rubber hand illusion [3] localized the brain areas responsible for feeling of ownership [2]. Ownership of a rubber hand activates areas in the premotor cortex, more specifically the left precentral sulcus and the right cerebellum. Moreover, using the RHI to create ownership and anxiety (by threatening the rubber hand with a needle) demonstrated that the stronger the illusion of ownership, the stronger the cortical anxiety response in insula and anterior cingulate cortex, and the higher the activation in the medial wall motor areas which reflect an urge to withdraw the hand [12]. Furthermore, [13] found that a physiological response, namely an increase in skin conductance, can be elicited by threatening a rubber hand during the RHI. Interestingly, ownership of an external body part is not limited to the hand. Recent studies, using the method of synchronous stroking of the body with a brush, have shown that a person's entire body can be projected to an external place [14], [15].

Based on these findings we hypothesize that measurable levels of ownership can be achieved through motor imagery, and could be influenced by verbal instruction. To test our hypothesis we created a scenario in which a virtual arm is threatened, and measured participants' physiological reactions to the threat. We measured both their galvanic skin response (GSR) and heart rate. Previous investigations have shown the galvanic skin response to be a sensitive measure of stressful situations in virtual environments [16]–[18]. The participants in our experiment were instructed to watch a video of a ball-interception game. The video showed two virtual arms that move and attempt to catch balls that appear to be moving towards the viewer. All participants had previously played the game themselves for one minute as part of another experiment [19]. Half of the participants only observed the video, while the other half observed and were instructed to imagine the two arms to be their own. During observation, the game was suddenly interrupted by a virtual “knife” that stabbed the virtual arm, which began to “bleed”. If our hypothesis is true, the observation-with-imagery group should show a stronger stress response, reflected by an increase of galvanic skin response and possibly by an increased heart rate, than the observation-only group.

## Results

The 23 subjects included in the study were all right-handed. All subjects except one correctly reported that no illumination changes occurred during the 87 second video. The subject who erred (he reported 3 changes for no obvious reason) was part of the “Observation only group”. His data did not differ in any significant way from those of others in the group and were thus included in the analysis. Subjects' behavioral reactions to the unexpected threat (i.e. vocal utterances such as “whoa” or “huh”, and/or smiling and laughing) suggested that they did not expect it to occur.

**Figure 1:**
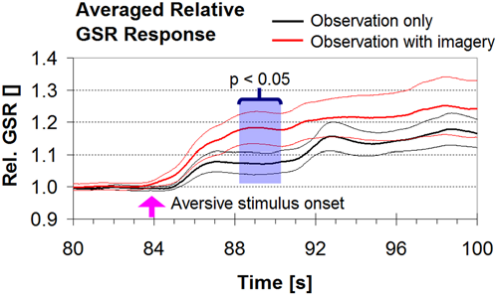
Averaged relative GSR for the two groups (red: observe and imagine, black: observe only). Thin lines indicate ±1 standard error. The blue shaded area indicates the time period (88.1–90.1 s) during which the observation-with-imagery group is significantly higher than the observation-only group (p<0.05). The time of onset of the aversive stimulus is shown as a violet arrow on the abscissa. The ordinate shows the averaged GSR relative to baseline, measured for 16 seconds prior to the onset of the aversive stimulus (see Methods).

Figure 1 shows the averaged GSR for each group, expressed relative to baseline (see Methods).

Prior to the appearance of the knife, the skin conductance was not significantly different between the two groups (t-test performed on the means for samples with unequal variance: p = 0.89; 7.2±3.7 µS [mean±standard deviation] for the “observation only group, 6.2±4.7 µS for the “observe and imagine” group). After the knife hit the virtual arm (at time 84±0.5 seconds, arrow on the abscissa) the skin conductance of both groups increased significantly above the baseline. In addition, there was a significantly higher increase of skin conductance for the observation-with-imagery group compared to the observation-only group during the interval between 88.1 and 90.1 seconds (t-test, one-tailed, p<0.05). This 2-second period corresponded to 62 consecutive time-points for which significantly different skin conductance levels were measured. The two curves differ maximally at 89.0 sec (p = 0.037). The curves then converged (i.e. they ceased to be statistically significantly different), but remained above baseline for several seconds after the end of the video. None of the other pairwise comparisons outside this 2-second period between showed a significant difference between the two curves.

Analysis of the heart rates did not show any significant change before and after the onset of the aversive stimulus, nor any significant change between the two groups.

After the experiment, subjects were given a questionnaire (see Methods). All responses were indistinguishable between the two groups, except for two statements. These were: the observation-only group enjoyed the task more (statement 7, t-test, two-tailed, p<0.05), and the observation-only group thought that the task was easier overall (statement 8, p<0.05). Of particular interest was the finding that the two groups were identical in their reported subjective “presence” in the task (statement 11, p>0.05) and their subjective ownership of the virtual arms (statement 3, p>0.05).

## Discussion

For motor rehabilitation systems using virtual reality tools [20], [21] it is important to know how bodily self-consciousness is achieved and extended to incorporate virtual objects. Are stimulation protocols such as the rubber hand illusion necessary to create a feeling of ownership of certain external objects, or can simpler methods achieve the same effect more easily?

The two groups showed statistically significant differences in their GSR following observation of an aversive stimulus. It is important to emphasize that the only difference between the two groups was the instruction to imagine the arms as being one's own. While it is fairly clear that the difference in the instructions caused the difference in the results, the question remains of whether the instructions caused the expected effect of mental imagery of the observed virtual arms. The possible confounds of the result include differences in the difficulty and enjoyment of the task, and a lack of between-groups differences in rating their subjective presence in the environment and ownership of the virtual arms.

A significant difference in subjects' attitudes was found in their rating of the ease of the task. The higher difficulty reported by the observation-and-imagery group seems to reflect the increased cognitive load involved in simultaneously watching the video and imagining ownership of the virtual arms. Although the virtual arms were oriented correctly from a first- person 3D perspective, they were represented on a flat screen and of course did not match the position of the subject's real arms. In addition, they were not very realistically rendered by modern computer graphics standards.

In addition to the increased perceived difficulty of the task, the observation-and-imagery group enjoyed the experiment less. This result could be due to the higher cognitive load of the task compared to the observation-only group, and/or increased ownership of the damaged virtual arm causing more negative responses to the virtual pain stimulus. We suggest that a combination of the two factors may contribute to the difference in enjoyment, but our data cannot isolate their relative contributions.

Although subjects' reported feelings of presence and ownership of the virtual arms was indistinguishable between the two experimental groups, the different increases of skin conductance suggests otherwise. This result could be related to the subjects' reported differences in task enjoyment and/or perceived difficulty.

A previous study found a significant within-subject correlation between subjectively reported increases in “fun” (i.e. enjoyment) and mean (baseline) GSR when switching from a less enjoyable to a more enjoyable task [22]. In their experiment they calculated mean GSR values for a computer game played over several minutes. The two populations in our study did not show a difference in mean baseline GSR levels *before* the aversive stimulus (see Results), suggesting (according to Mandryk and Inkpen) that our pre-aversive-stimulus *baseline* tasks (observation alone vs observation with imagery) were probably indistinguishable in terms of enjoyment. Since the reported enjoyment was not dependent on the baseline period, the dependence of GSR on enjoyment in our experiment must have been due entirely to the differentiated response to the aversive stimulus caused by the two different tasks. The difference was invisible during the baseline period but manifested itself when the aversive stimulus occurred. Thus, the tasks were not more or less enjoyable per se, but rather the lower reported enjoyment could have been caused by the combination of increased ownership with the aversive stimulus. Interestingly, our inverse transient post-stimulus relationship between fun and GSR was the opposite of that found for baseline enjoyment by Mandryk and Inkpen. We suggest tentatively that the difference may be related to the key GSR measurement in Mandryk and Inkpen (2004) reflecting an arousal-driven baseline, while in our case the GSR measurement reflected a transient aversive response to a stimulus.

With respect to the influence of task difficulty, at least one study of skin conductance during a simple perceptual-motor task found no dependence of the mean skin conductance on task difficulty [23]. Given that task enjoyment and difficulty are unlikely to have affected our results and that the reported ownership was indistinguishable between the groups, we suggest that the induced feeling of ownership may be at least partially subconscious. It is not clear whether this subconscious form of ownership is of a different nature than that induced by the rubber hand illusion, or a weaker form of the same type of ownership.

Other studies measuring subjective presence using galvanic skin response, heart rate variability or skin surface temperature usually employed stronger illusions or more highly anxiety-inducing situations compared to our experiment. In a study of the rubber hand illusion using a physical rubber hand and visuo-tactile stimulation, different levels of ownership were created and anxiety was induced by threatening to hurt the rubber hand with a needle [13]. In agreement with our results, the authors showed a significant increase of skin conductance in conditions when ownership was experienced compared to those which evoked little or no ownership. In an fMRI study using the rubber hand illusion [12], a correlate of the cortical anxiety response to threats was found. The anxiety response was similar for the rubber hand with ownership and threats to the participants' real hand, but was much smaller when the rubber hand was threatened during a no-ownership condition. Similar studies using virtual environments have evoked physiological responses by exposing subjects to vertigo-inducing pits [16], [24] or individual phobias such as public speaking [17], [18]. A virtual reality reprise of the famous Stanley Milgram experiments has also been conducted, in which test subjects administer virtual electroshocks to virtual characters when they answer questions incorrectly [25]. The subjects' elevated physiological reactions and questionnaire responses suggested high stress levels, indicating that humans can relate not only to real [26] but also to virtual pain. Compared to these studies, our stimuli were not highly immersive and we used a much simpler stimulation protocol, but our results were nevertheless consistent with those of the other studies.

Several current theories of bodily self-consciousness suggest that it may be achieved through multi-modal Bayesian perceptual learning or some bottom-up mechanisms combined with cognitive constraints [1], [13], [15]. The result of our experiment, including a single sensory modality (except for the brief sound associated with the onset of the aversive stimulus), suggests that body ownership does not necessarily require multi-modal input to be elicited. Rather, we suggest that a combination of bottom-up sensory input and imposed top-down mental imagery can induce ownership which is similar in strength to multi-modal input.

In our experiments, it is unclear whether the lack of photo-realism of our virtual arms was a help or a hindrance for inducing ownership. Skin conductance responses and activation of brain areas related to emotion and attention (as measured by fMRI) can be related to very abstract stimuli such as winning or losing small amounts of virtual money [27]. While higher realism might seem intuitively better for inducing ownership, the “uncanny valley” hypothesis suggests that realistic but obviously not real graphics could be disturbing for inducing humans to see a virtual environment as real [28]. A recent result in support of this hypothesis as applied to ownership was found in virtual reality out-of-body experiments [15]. In these experiments, a larger proprioceptive drift towards the virtual body was found when the extracorporal body was not a projection of the participants' actual body, but the projection of a fake body.

The fact that we did not find a significant increase in heart rate, despite the clear change in skin conductance, could be due to a number of factors. As real acute pain does not necessarily increase heart rate [29], it is possible that the results are entirely consistent with the perception of acute virtual pain. Alternatively, three attributes of the stimulus may have combined to produce the result: its virtual nature, its low realism, and its short duration. A more realistic representation of the arm and knife, or a stimulus of longer duration such as a lingering threat to hurt the virtual arm without inflicting virtual pain, may produce a measurable change in heart rate.

Our experiment shows that the instruction to imagine ownership of part of an observed video measurably modulates physiological responses to aversive stimuli. Potential applications of this finding include situations in any virtual reality system where eliciting ownership is required, such as gaming and rehabilitation.

It is currently unknown to what extent the underlying cortical mechanisms of ownership we postulate to be recruited in our experiment overlap with those elicited using multimodal stimulation such as the true rubber hand illusion. Neuroimaging methods, e.g. fMRI, could be applied to determine the detailed differences between the two stimulation methods. While it is likely that our stimulus method will elicit similar premotor cortex activation to that seen in the rubber hand illusion, the activation may be weaker in our case as the subjects' questionnaire responses suggest that our ownership effect is at least partly subconscious. The effect of auditory stimulation is another area for future investigation. All game-related events in the video had an associated sound effect. Our animated knife also had an accompanying sound effect, similar in volume and length to the preceding ball-related sound effects. Future investigations could focus on omitting the knife sound effect, the other sound effects and replacing them with more realistic sounds. Our stimulation protocol differed somewhat to that in [12], [13]: we did not just threaten the incorporated external objects, we also actually (albeit virtually) “hurt” it. It is unknown whether threatened virtual pain, rather than actual virtual “pain”, would lead to a higher or lower physiological response. We suggest that because the threat of virtual pain can be applied over a longer time than actual virtual pain (where the subject quickly realizes that there is no real physical pain), it may be more likely to produce slower but measurable heart rate changes for our stimulation method.

## Materials and Methods

### Participants

A total of 23 subjects (12 observation only [9 male/3 female], 11 observation with imagery [8 male/3 female]) were recruited using flyers and word-of-mouth propaganda on two campuses of the ETH Zürich and the University of Zürich. The participants' ages ranged from 20 to 45 years (mean age 25.0 years; std. dev. 2.9 years). Prior to the start of the experiments, participants' handedness was assessed using the Edinburgh Handedness Inventory [30] to determine the non-dominant hand for the skin conductance measurements. The experiments were conducted in a single individual session of about 45 minutes for each subject, for which they were paid the equivalent of USD 20. All participating subjects gave written informed consent prior to the experiments and signed a receipt for having received remuneration at the end of the session. The written and verbal instructions were provided in the subject's choice of English or German. All procedures were approved by the ethics committee of the ETH Zurich.

### Technical set-up

Participants were seated comfortably at a desk on a height-adjustable chair in a quiet room, about 70 cm in front of a flat LCD TV monitor (Acer, 90 cm diagonal, 1366×768 pixels). The monitor was connected to a PC (Dell OptiPlex 320, Celeron 3.06 GHz, 1 GB RAM). The video was recorded directly from the screen at high resolution using SnagIt (TechSmith, USA).

### Procedure

**Figure 2:**
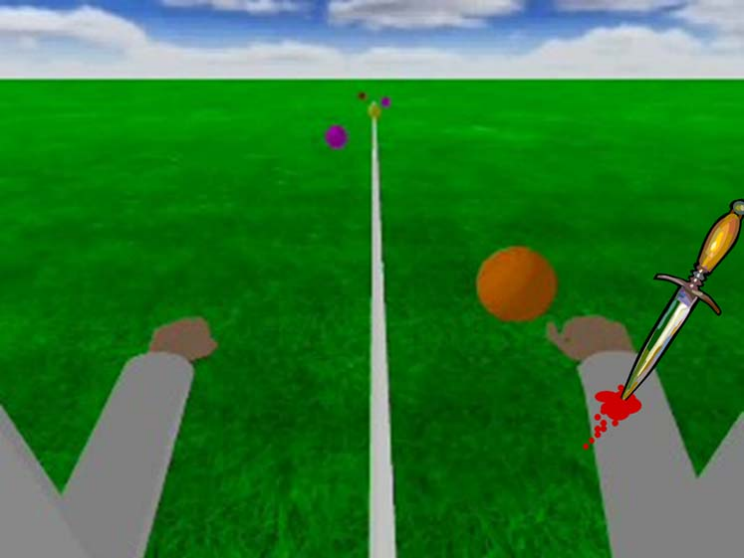
Subject stimulus view on screen, showing virtual arms, incoming virtual balls and virtual injury caused by a “knife”.

The experiment was based on a simple interactive computer game [31]. In the real game, players have a first-person perspective view of two arms and a large green field (Figure 2). Different colored balls appear in the far distance and move in a straight line towards the player, along a trajectory parallel to the centerline. When playing the game, players wear data gloves with built-in digital compasses, and intercept the balls by moving the virtual arms. In addition to visual feedback, success or failure for each ball is indicated by different sounds.

Participants were randomly assigned to one of two groups. All participants played a simplified version of the game for one minute before the start of the experiment. In the simplified game, subjects simply had to move their arms freely and observe the corresponding on-screen virtual arm movements; no balls appeared for them to intercept.

For the experiment proper, all subjects watched the same pre-recorded video (duration 87 seconds) of a game played by another person (also right-handed). While watching the video, subjects rested their arms on the table with their palms facing downwards on the table. They were instructed to not move their arms while watching the video. One group, the “observe only” group, was instructed to watch the video by the following on-screen instructions:

- Watch the screen. You will see a video of two arms playing a ball-hitting game.
- While you are watching, note how many times the global illumination changes.
- At the end of the task, report how many times the global illumination changed.

The other group, “observe and imagine”, received the same instructions as above but with the following additional instruction in between the first and second lines:

- Concentrate on the video. Imagine that the arms on the screen are *your* arms.

To ensure that participants concentrated on the video, they were also told to count changes in global illumination that occurred during the video. In fact, no illumination change occurred at all. Three seconds before the end of the video, a virtual “knife” flew in from the right side of the screen and stabbed the right virtual arm and immediately started to “bleed”, and then remained stationary on the screen after the end of the video. The stabbing of the arm was accompanied by a brief “whooshing” and “punch” sound.

### Physiological measures

Skin conductance and heart rate were measured using biofeedback hardware (Wild Divine, Colorado, USA) attached to the index-, middle- and ring-finger of the participant's non-dominant hand. The skin conductance and heart rate recordings were started manually synchronously with the start of the video, with an accuracy estimated as plus or minus 0.5 seconds. The skin conductance measurements were calibrated in the laboratory against standard resistances known to an accuracy of 0.01%. The data was sampled every 32 ms, i.e. at 31.25 Hz. Recording started at the beginning of the video, and continued for 16 seconds following the onset of the aversive stimulus.

### Statistical Analysis

We smoothed the GSR data with a median filter over 10 values (i.e. over 9 time intervals corresponding to a window of 289 ms). A baseline was taken as the average of the skin conductance over a 16-second interval ending when the knife hit the virtual arm. Changes were calculated as percentage increases from the baseline. The beat-by-beat heart rate data was smoothed with a median filter over 49 values (about 1.6 seconds).

Statistical analysis was performed using t-tests with unequal variance at a 5% significance level. To compare the GSR traces for the two test conditions, we performed t-tests at each recorded time-point within a 16-second baseline window before the aversive stimulus onset (500 points at 31.25 Hz), and also for each time-point during a 16-second period after the aversive stimulus onset (another 500 points at 31.25 Hz). During the 16-second period after the aversive stimulus onset, the video was still running for the first three seconds (i.e. 94 data points while showing the bleeding arm), followed by 13 seconds (i.e. 406 data points) with a stationary image of the “injured” arm. To define the epoch where the curves diverged significantly from each other, we took the first and last points where the curves were significantly different. The multiple comparisons correction factor (e.g. Bonferroni) for the epoch can then be defined as one plus the number of points within the epoch where p>0.05. Using this approach accounts for the potential problem of multiple comparisons reducing the power of the statistical test; see this method also used e.g. in [32] figures 6 and 7.

### Questionnaires

After completing the task, all participants answered a questionnaire with eleven items (Table 1). Responses to each statement (except the first one) were given on a scale from one to seven, with one indicating strong disagreement and seven indicating strong agreement. In statements 7, 8 and 11 the word “task” is used to refer to the observation or observation-plus-imagery. To prevent possible confusion with the other “task” (counting illumination changes), it was made clear during subject recruitment and instruction that the primary “task” at hand was an observation or an observation-plus-imagination task. In the unlikely case that subjects were confused about the statements, there is no reason to believe that this confusion would be more prevalent in one group or the other.

**Table 1:** Subject questionnaire statements.

#	Statement
1	How many times did the global illumination change? [numerical response]
2	The virtual arm was realistically rendered.
3	While watching the video, I forgot that the virtual arm was not my own.
4	When the arm was hit, I felt the urge to retract it.
5	I am experienced with playing computer games in general.
6	I am experienced with playing first-person “ego shooter” computer games.
7	I enjoyed the task.
8	The task was easy.
9	I was shocked by the knife.
10	I am generally rather jumpy.
11	I felt immersed in the task.
